# Optimizing word embeddings for small datasets: a case study on patient portal messages from breast cancer patients

**DOI:** 10.1038/s41598-024-66319-z

**Published:** 2024-07-12

**Authors:** Qingyuan Song, Congning Ni, Jeremy L. Warner, Qingxia Chen, Lijun Song, S. Trent Rosenbloom, Bradley A. Malin, Zhijun Yin

**Affiliations:** 1 Vanderbilt University, Nashville, TN 37240 USA; 2 Brown University, Providence, RI 02903 USA; 3 Vanderbilt University Medical Center, Nashville, TN 37232 USA; 4 Rhode Island Hospital, Providence, RI 02903 USA

**Keywords:** Breast cancer, Hormonal therapy, Natural language processing, Patient portal messages, Word embedding models, Word2vec, Machine learning, Breast cancer

## Abstract

Patient portal messages often relate to specific clinical phenomena (e.g., patients undergoing treatment for breast cancer) and, as a result, have received increasing attention in biomedical research. These messages require natural language processing and, while word embedding models, such as word2vec, have the potential to extract meaningful signals from text, they are not readily applicable to patient portal messages. This is because embedding models typically require millions of training samples to sufficiently represent semantics, while the volume of patient portal messages associated with a particular clinical phenomenon is often relatively small. We introduce a novel adaptation of the word2vec model, PK-word2vec (where PK stands for prior knowledge), for small-scale messages. PK-word2vec incorporates the most similar terms for medical words (including problems, treatments, and tests) and non-medical words from two pre-trained embedding models as prior knowledge to improve the training process. We applied PK-word2vec in a case study of patient portal messages in the Vanderbilt University Medical Center electric health record system sent by patients diagnosed with breast cancer from December 2004 to November 2017. We evaluated the model through a set of 1000 tasks, each of which compared the relevance of a given word to a group of the five most similar words generated by PK-word2vec and a group of the five most similar words generated by the standard word2vec model. We recruited 200 Amazon Mechanical Turk (AMT) workers and 7 medical students to perform the tasks. The dataset was composed of 1389 patient records and included 137,554 messages with 10,683 unique words. Prior knowledge was available for 7981 non-medical and 1116 medical words. In over 90% of the tasks, both reviewers indicated PK-word2vec generated more similar words than standard word2vec (p = 0.01).The difference in the evaluation by AMT workers versus medical students was negligible for all comparisons of tasks’ choices between the two groups of reviewers (${\text{p}} = 0.774$ under a paired t-test). PK-word2vec can effectively learn word representations from a small message corpus, marking a significant advancement in processing patient portal messages.

## Introduction

Patient portals have become standard components of modern electronic health record (EHR) systems^1^. They enable patients to access health information, manage clinical appointments, and communicate securely with care providers in an asynchronous manner^2,3^. The messaging functionality of patient portals has gained in popularity over time^4,5^ due to various benefits, such as the ability to increase patient involvement in medical decision-making^6^. At the same time, the information conveyed in patient portal messages can serve as a foundation for clinical research, such as studies of initiation and discontinuation of cancer therapies^7,8^, assessing readmission risk^9,10^, and creating taxonomies of patient engagement^11^.

However, maximizing the utility of patient portal messages requires a means to extract meaningful signals from unstructured text^12^. Rule-based methods, which rely on manually pre-defined rules to perform a task, are ineffective as the volume of data or complexity of the task increases^12^. While specific computational methods, such as topic modeling, can be applied in this regard, these approaches typically rely on word frequency to determine the most representative words for each topic. By contrast, word embedding models, such as word2vec^13^, have been widely adopted by natural language processing (NLP) applications. These models can learn vector representations, typically from millions of documents, to capture each word’s semantic and linguistic meaning. However, it is challenging to procure a cohort that is sufficiently large to train a specific word embedding model. As an illustration, in this paper, we focus on breast cancer as it serves as an exemplar of a complex care coordination setting between various specialists who often communicate with patients via the patient portal^14^.

The current approaches for generating word embeddings on relatively small corpora may learn semantic meaning inaccurately due to lack of sufficient occurrences of word usage. A natural solution to this problem is to fine-tune a word embedding model that is pre-trained on a larger data corpus. This is because the fitted parameters of a pre-trained model can serve as a better starting point for training than random initialization. Yet this strategy presents certain limitations as well. In particular, pre-trained models are less accurate when handling words that are absent from the pre-trained model’s vocabulary^15^. Moreover, inconsistencies arise due to the incompatible linguistic contexts between the data used to fit the pre-trained model and the data for a specific domain of interest^16^.

In this study, we introduce a novel approach, PK-word2vec (where PK stands for prior knowledge), to train word2vec models on a patient portal message corpora of relatively small sample size. Our model leverages prior information, as defined by the most similar words sampled from pre-trained embedding models, to regularize the model training. We demonstrate the utility of this approach as a case study using a dataset of approximately 137,000 patient portal messages from 1389 patients diagnosed with breast cancer at a large academic medical center. Our experiments show that the regularization process of PK-word2vec can guide and constrain the model training to learn word semantic meanings more effectively than standard word2vec models. By applying this methodology, researchers can achieve higher quality word embeddings with limited data, which is particularly advantageous for rare medical terms and conditions. Additionally, in clinical care, the improved embeddings can enhance natural language processing applications such as patient-provider communication analysis, which has potential to improve patient outcomes through more accurate and nuanced understanding of patient language.

## Methods

**Figure 1:**
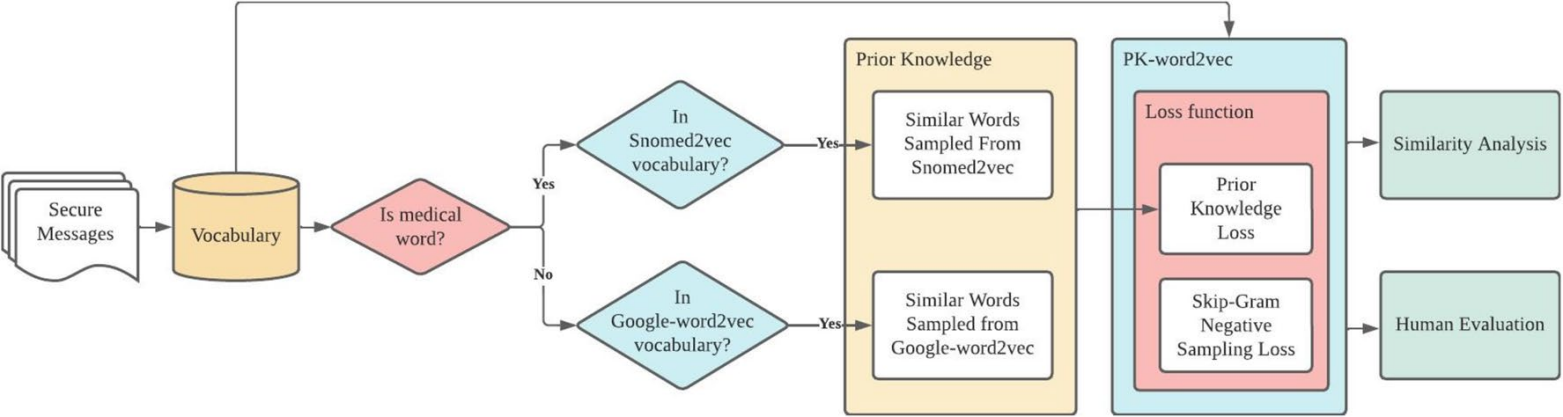
Workflow associated with the creation of the PK-word2vec model.

Figure 1 depicts the workflow for this study: (1) data extraction and cleaning; (2) prior knowledge extraction from two pre-trained models, Snomed2vec^17^ and the Google pre-trained word2vec (Google-word2vec)^13^ for medical and non-medical words, respectively; (3) model training regularized by prior knowledge (PK-word2vec); and (4) similarity analysis and human evaluation of the model performance.

### Data collection and preprocessing

We collected the patient portal messages from the EHR system of Vanderbilt University Medical Center (VUMC), a large, private, non-profit, academic medical center in Nashville, Tennessee. VUMC launched its patient portal, *My Health at Vanderbilt* (*MHAV*), in 2004, which was migrated to Epic’s MyChart platform in 2017. MHAV supports secure messaging between healthcare providers and patients, appointment scheduling, billing management, and access to laboratory results or other EHR data^5^. This study was deemed exempt from human subjects research by the Vanderbilt University Institutional Review Board.

In this study, we focused on the patient portal messages for patients with breast cancer who were prescribed the most common hormonal therapy medications: *anastrozole*, *exemestane*, *letrozole*, *raloxifene*, and/or *tamoxifen*. We masked URLs, email addresses, phone numbers, timestamps, and numerals by using the *ekphrasis* (v0.5.1)^18^. Afterwards, all personal names and usernames were manually masked, and misspelled words were corrected with the guidance of two domain experts (STR, JLW) by referring to the contexts from randomly selected messages that contain these misspelled words. We applied the CLAMP software package (v1.6.4) to detect medical words pertaining to diseases, laboratory tests, and medications from messages^19^.

### Skip-gram model with negative sampling

**Figure 2:**
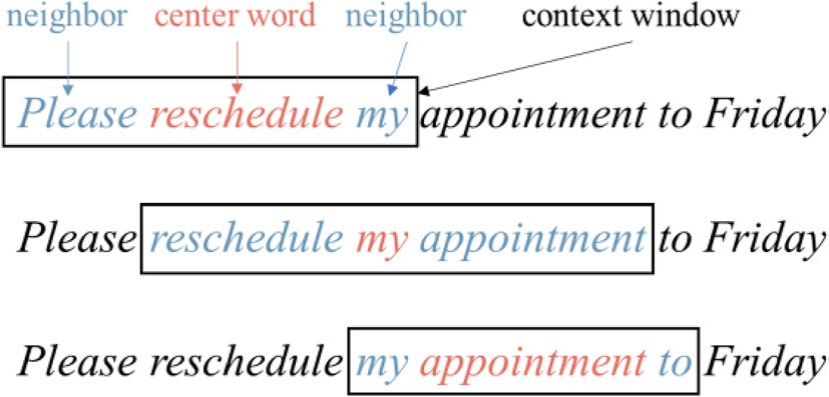
An illustration of a context window with a size of 1, a center word and its neighbors in the SGNS framework.

PK-word2vec is built on a skip-gram model with negative sampling (SGNS)^13^, a standard word2vec approach commonly used for creating word embeddings. In SGNS, each word has two vector representations: (1) a center vector when it is processed as a center word, and (2) a context vector when it serves as a neighbor of a center word. Figure 2 illustrates the center word and neighbor word within a context window size of 1 in the sentence “*Please reschedule my appointment to Friday*”. In this example, *reschedule* is one of the neighbors of the center word *appointment*. Formally, SGNS structures the model training as a binary classification problem, where the objective is to predict whether a certain pair of words are neighbors. Formally, given a sequence of words $w_{1} , w_{2} , \ldots , w_{T}$ and a context window of size $m$, the SGNS objective function is defined as:

1 $$Loss_{SGNS} = \mathop \sum \limits_{t = 1}^{T} \left[ {\mathop \sum \limits_{ - m \le j \le m} \left[ { - \log \sigma \left( {{\mathbf{u}}_{t + j}^{T} {\mathbf{v}}_{t} } \right) - \mathop \sum \limits_{{k = 1, w^{\prime}_{k} \sim P\left( {w_{t} } \right)}}^{K} \log \sigma \left( { - {\mathbf{u}}_{k}^{T} {\mathbf{v}}_{t} } \right)} \right]} \right]$$

where ${\mathbf{u}}_{t}$ and ${\mathbf{v}}_{t}$ represent the context and center vector of $w_{t}$, which are the parameters optimized in the loss function; $w^{\prime}_{k}$ represents a random word that does not appear in the neighboring context of $w_{t}$. $w^{\prime}_{k}$ is sampled from $P\left( {w_{t} } \right)$, a unigram distribution defined by the frequency of occurrence of each word^20^; $K$ is a hyperparameter that specifies the number of negative words sampled for $w_{t}$; and $\sigma$ is the sigmoid function. The first term in Eq. (1) aims to maximize the probability of occurrence for words that are in the context window. By contrast, the second term in Eq. (1) aims to minimize the probability of occurrence for words that are not in the context window.

### Prior knowledge Word2vec model

The challenge of training a word2vec model on a small dataset arises from the insufficient context that such datasets provide. To address this issue, we define the prior knowledge of a word as its most similar words identified from other pre-trained models. Subsequently, the prior knowledge is applied to regularize the PK-word2vec model training. More formally, if a word in the patient portal messages $w_{t}$ also appears in a pre-trained embedding vocabulary, the prior knowledge of $w_{t}$ is defined by another word $w_{j}$ that is sampled with probability $p_{tj}$ from the patient portal message vocabulary. In this respect, the more similar $w_{j}$ is to $w_{t}$ in the pre-trained embedding model, the greater the likelihood that $w_{j}$ will be sampled.

We introduce a prior knowledge loss function for the entire dataset, which is defined as follows:

2 $${\text{Loss}}_{{{\text{PK}}}} = \left\{ {\begin{array}{*{20}c} {\mathop \sum \limits_{t = 1}^{T} \left[ {\gamma \left( {w_{t} } \right) \cdot {\Psi }\left( {{\mathbf{u}}_{t} ,{\mathbf{u}}_{j} ,{\mathbf{v}}_{t} ,{\mathbf{v}}_{j} } \right)} \right]} & { w_{t} {\text{with prior knowledge}}} \\ 0 & {w_{t} {\text{without prior knowledge}}} \\ \end{array} } \right.$$

where $\gamma \left( {w_{t} } \right) = \tau /{\text{freq}}\left( {w_{t} } \right)$ discounts the words proportionally to their frequency, $\tau$ is a hyperparameter, and ${\Psi }\left( \cdot \right)$ is a regularization factor to be defined in Eq. (4) below. Note that if a word does not exist in the pre-trained model, then it has no prior knowledge and its corresponding term in ${\text{Loss}}_{{{\text{PK}}}}$ is set to zero.

In this work, we calculate word similarity as the cosine similarity of two vectors ${\mathbf{u}}$ and ${\mathbf{v}}$, $\cos \left( {u,v} \right) = u \cdot v/\left( {\left| u \right| \cdot \left| v \right|} \right)$. We require that each sampled $w_{j}$ have a similarity score with $w_{t}$ above a pre-defined threshold $\theta$. As such, $p_{tj}$ is defined as:

3 $$p_{tj} = \left\{ {\begin{array}{*{20}c} {{\text{softmax}}\left[ {\cos \left( {{\mathbf{u}}_{t} ,{\mathbf{u}}_{j} } \right)} \right]} & {\cos \left( {{\mathbf{u}}_{t} ,{\mathbf{u}}_{j} } \right) \ge \theta } \\ 0 & {\cos \left( {{\mathbf{u}}_{t} ,{\mathbf{u}}_{j} } \right) < \theta } \\ \end{array} } \right.$$

In our experiments, for each pre-trained model, we set $\theta$ to the average of the top 10 similarity scores for words with prior knowledge in patient portal messages. In practice, each time $w_{t}$ is processed in model training using gradient descent optimization, a word $w_{j}$ is sampled and its cosine similarity with $w_{t}$ in both context and center vectors is calculated as its prior knowledge. Then, the corresponding item for $w_{t}$ that needs to be minimized in the PK-word2vec loss function in Eq. (2) is:

4 $${\Psi }\left( {{\mathbf{u}}_{t} ,{\mathbf{u}}_{j} ,{\mathbf{v}}_{t} ,{\mathbf{v}}_{j} } \right) = \left[ {1 - \cos \left( {{\mathbf{u}}_{t} ,{\mathbf{u}}_{j} } \right)} \right] + \left[ {1 - \cos \left( {{\mathbf{v}}_{t} ,{\mathbf{v}}_{j} } \right)} \right]$$

There are several reasons for incorporating context vector similarity into Eq. (4). First, a key characteristic of high-quality word embeddings is that the distance between the context vectors for similar words is relatively small^21^. Second, since the context vectors are applied to estimate the center vectors of neighboring words, the regularization of the context vectors can propagate to the center vectors of neighboring words without prior knowledge. Finally, the overall objective function for PK-word2vec can be defined as:

5 $${\text{Loss}} = {\text{Loss}}_{{{\text{SGNS}}}} + \alpha \times {\text{Loss}}_{{{\text{PK}}}}$$

where $\alpha$ is a hyperparameter that determines the degree to which the training of PK-word2vec relies on prior knowledge. A higher $\alpha$ indicates a stronger dependency on prior knowledge. To evaluate the impact of α, we compared our approach to a baseline model where $\alpha =0$, a condition corresponding to SGNS. We generate prior knowledge for non-medical and medical words using two different models. Specifically, we applied Google-word2vec, which was trained on the Google News dataset (about 100 billion words), to build prior knowledge for non-medical words. We turned to a *Snomed2vec* model, which was trained on the SNOMED-CT knowledge graph^17^ to obtain prior knowledge for medical words.

### Hyperparameter selection

Hyperparameter tuning for word2vec models is challenging because it is difficult to define similarity. Since Google-word2vec was trained on a vast corpus, we selected hyperparameters, the dimension of word embedding *d* and the prior knowledge weight $\alpha$, such that PK-word2vec is similar to Google-word2vec in terms of the distribution of the similarity scores between a given word and its 10 most similar words. In this paper we name this distribution as similarity distribution for simplicity. We did not use Snomed2vec as a reference because it is inherently a node2vec^22^ trained on a semantic network in the Unified Medical Language System^23^ rather than text data.

We set the embedding size $d$ with a value that corresponds to the smallest Wasserstein distance^24^ in the similarity distributions between the two models. To select $\alpha$, we first categorized words into high-, mid-, and low-frequency ranges based on their tertiles. Then, we set $\alpha$ with a value that corresponds to the smallest Wasserstein distance in the similarity distribution between three distinct word frequency ranges. This resulted in a comparison in an unbiased manner, since high-frequency words tended to receive better representations. When training PK-word2vec, the $\theta$ threshold was set to the average word similarity between all the words and their 10 most similar words in the pre-trained models. We set the threshold $\tau$ in Eq. (2) to 0.00001, batch size to 10,000, and trained the model for 10 epochs.

### Human evaluation

To compare PK-word2vec with SGNS, we randomly sampled 1,000 words from the patient portal message vocabulary, with the same proportion of medical to non-medical words. For each term of interest, we created one group of the five most similar words generated from PK-word2vec and SGNS, respectively. To evaluate the system, we asked each reviewer to indicate which word group was more related to the word of interest. We randomized the order of the two groups in each task to avoid framing biases.

We recruited two groups of reviewers for the model evaluation. The first group consisted of 330 Amazon Mechanical Turk (MTurk) workers^25^, recognized as “masters” due to their consistently submission of high-quality results in their history annotations. Each MTurk worker completed 100 out of the 1000 tasks and each task was answered by 33 different MTurk workers. The second group consisted of 7 medical students recruited through the Vanderbilt Institute for Clinical and Translational Research. Each medical student completed all 1000 tasks. We set a 30-s time limit for each task; task not completed within this time limit were reassigned to a new worker.

To analyze the human evaluation data, we first calculated the support rate, defined as the proportion of the 1,000 tasks for which MTurk workers or medical students preferred PK-word2vec by a majority vote. Next, we employed sample skewness^26^ to examine the distribution of the proportion of reviewers who favored PK-word2vec in each task. This provided insights into the extent to which PK-word2vec was favored over SGNS. A larger value for left sample skew indicates a preference of PK-word2vec over SGNS. Finally, to account for the heterogeneity, we fitted a mixed-effects logistic regression model to assess which word embedding model was preferred by MTurk workers. The type of model (PK-word2vec or SGNS) and the category of the term of interest (medical or non-medical words) were variables with fixed effects. We assigned a value of 1 to the PK-word2vec model and medical words and a value of 0 to the SGNS and non-medical words in the corresponding variables.

## Results

### Data summary

The dataset was composed of 137,554 messages sent by 1389 patients with breast cancer. After preprocessing, there was a total of 10,683 unique words in the vocabulary, of which 8878 (80%) and 1895 (20%) were non-medical and medical words, respectively. Prior knowledge was available for 7981 (90%) non-medical and 1116 (59%) medical words.

### Hyperparameter selection

#### Embedding size ($d$)

**Figure 3:**
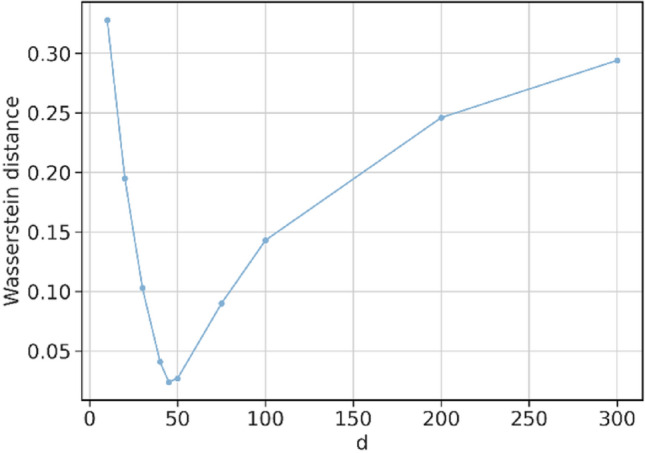
The Wasserstein distance in the similarity distribution between Google-word2vec and SGNS as a function of embedding size. A sensitivity analysis to optimize the word embedding size.

The word embedding size was selected from a range of 10–300. Figure 3 depicts the Wasserstein distance between SGNS and the Google-word2vec as a function of the word embedding size. In the following experiments, we present results with an embedding size of 45 for SGNS and PK-word2vec because it corresponds to the smallest distance among all the candidate vector sizes.

#### Prior knowledge weight ($\alpha$)

**Figure 4:**
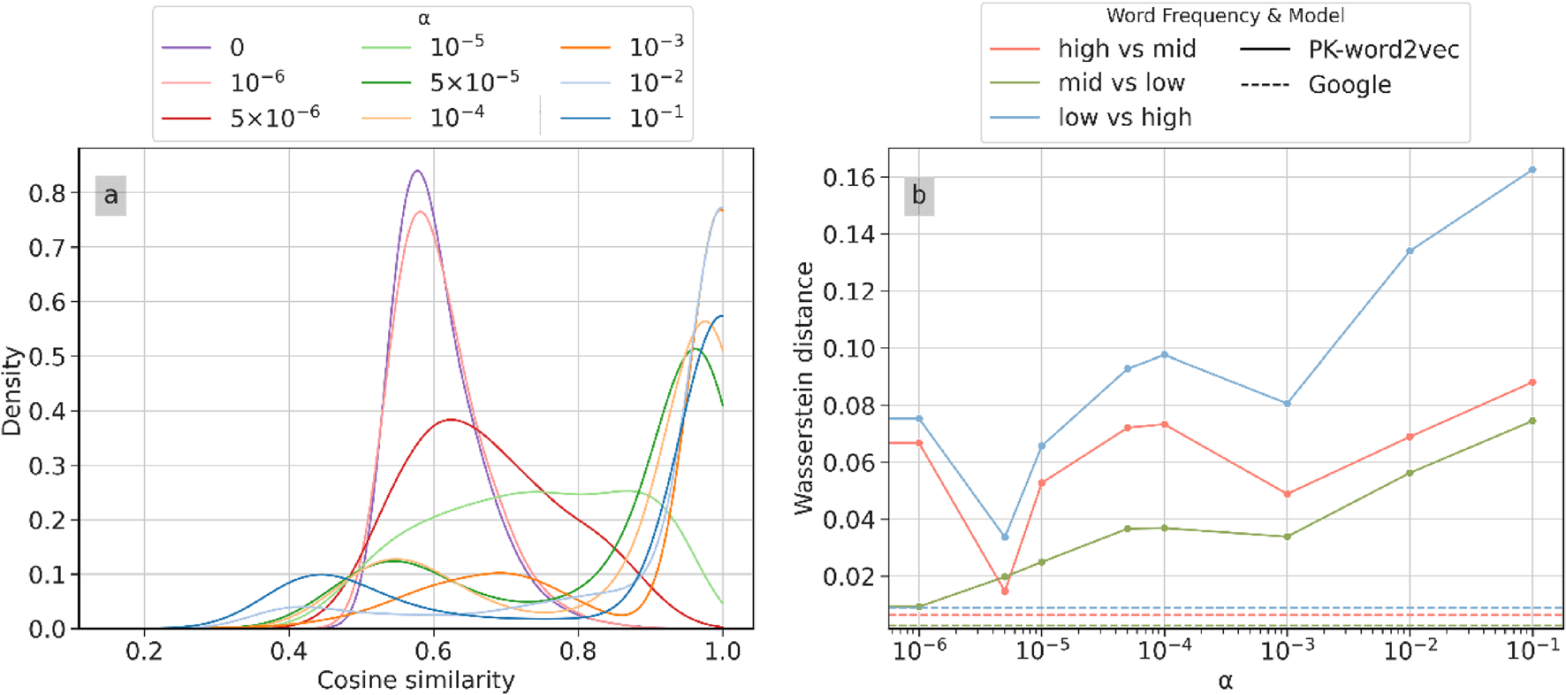
Sensitivity of similarity distribution (left) and Wasserstein distance between Google-word2vec and PK-word2vec on various word frequencies (right) across the range of $\alpha$ values.

Figure 4a shows the similarity distribution for PK-word2vec as a function of $\alpha$. When $\alpha = 0$, this model corresponds to SGNS and most of the density concentrates around 0.6. As $\alpha$ increases, the prior knowledge becomes dominant, and the density gradually shifts toward two directions, forming two peaks. Figure 4b shows the Wasserstein distance in the similarity distribution between Google-Word2vec (three dashed lines at the bottom of the figure) and PK-word2vec (three solid lines at the top of the figure) in different word frequency ranges. The distributions for Google-word2vec were nearly flat and maintained relatively small values. Based on this observation, we set $\alpha$ to $5 \times 10^{ - 6}$ for the following experiments because it corresponds to the smallest Wasserstein distances as well as smallest variance across three pairs of comparisons.

### Word similarity

#### Context vector regularization

**Figure 5:**
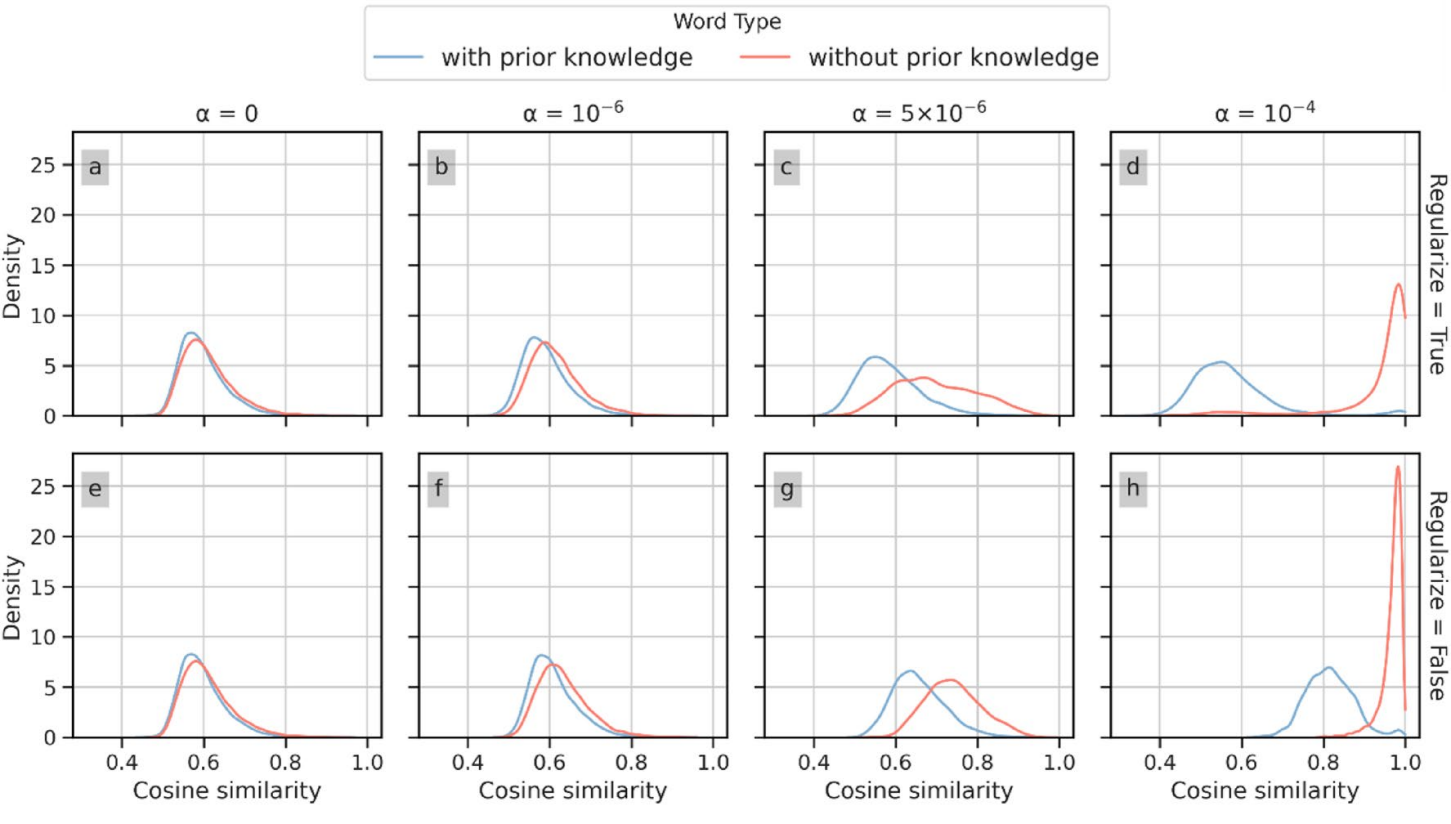
The distribution of the words with the 10 highest similarity scores of words with and without context vector regularization during model training.

Figure 5 depicts the similarity distribution for the models with (panels a–d) and without (panels e–g) context vector regularization. The blue and orange lines in each graph correspond to words with and without prior knowledge, respectively. The comparisons between two similarity distributions are illustrated in settings with different pre-selected $\alpha$ values. As extreme examples, Fig. 5d and g show that when $\alpha = 10^{ - 4}$, context vector regularization mitigates the skew of the similarity distribution for the words with prior knowledge. While a large $\alpha$ value shifts the mode of the similarity distribution for words without prior knowledge in the model without context vector regularization to the right, the variance of similarity distribution is not significantly altered. Figure 5c and f depict the scenario where $\alpha$ is set to its optimal value of $5 \times 10^{ - 6}$, where the difference between the two distributions is in an intermediate state compared with other $\alpha$ values. These results show that context vector regularization mitigates the impact of prior knowledge on the similarity distribution between words with and without prior knowledge.

### Human evaluation

**Figure 6:**
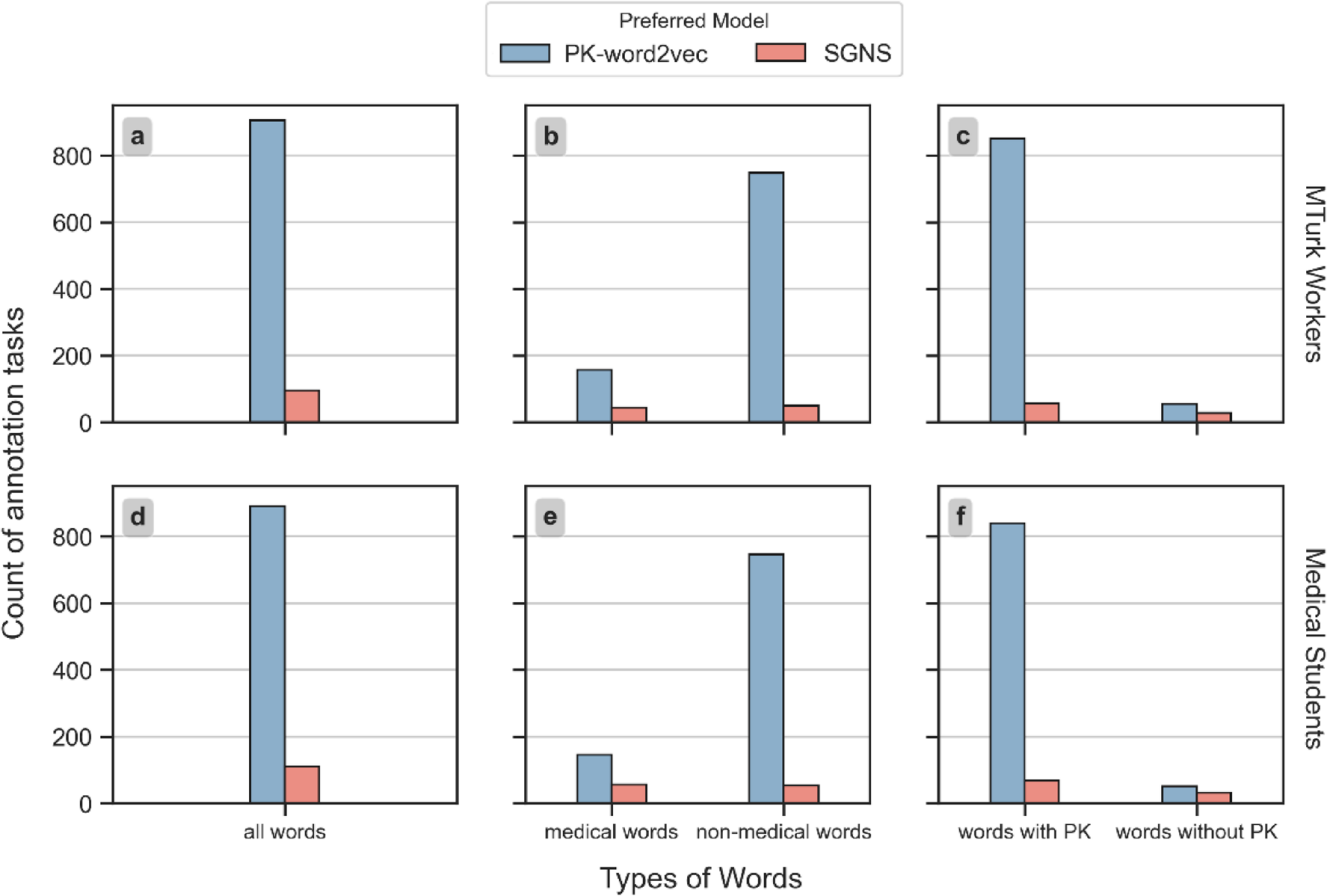
The number of tasks where one model was preferred over the other based on a majority vote by MTurk workers (upper row) and medical students (lower row).

Figure 6 displays the human evaluation results based on a majority vote from the MTurk workers and medical students. In addition to an overall comparison, the results were stratified by the word type, and the existence of prior knowledge. The difference was negligible for all comparisons between the two groups of reviewers (${\text{p}} = 0.774$ under a paired t-test). In over 90% of the tasks, both groups of reviewers indicated that PK-word2vec generates more similar words than SGNS. For medical words, the support rate for PK-word2vec was 78.8% for MTurk workers and 71.8% for medical students. For non-medical words, the support rate for PK-word2vec was 93.6% for MTurk workers and 93.4% for medical students. For words lacking prior knowledge, the support rate for PK-word2vec was 66.7% from MTurk workers and 61.4% from medical students.

**Figure 7:**
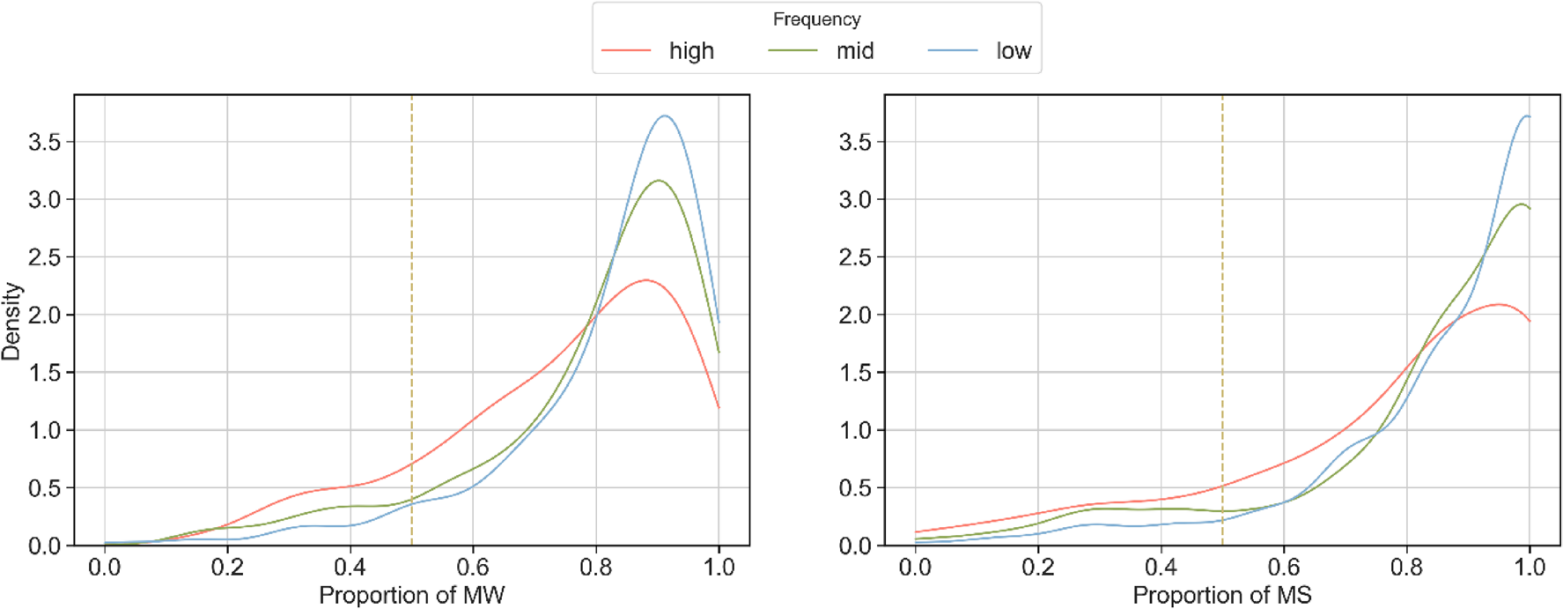
Distribution of the proportion of reviewers that choose PK-word2vec over SGNS per annotation task (n = 1000). Different colors indicate the word frequency ranges of the original word in each task. The vertical dashed line corresponds to 0.5, which indicate that there was no difference in the annotator preference.

Figure 7 shows the distribution of the proportion of reviewers that favor PK-word2vec in each task. The three curves represent the proportion distribution for the low-, mid-, and high-frequency words, respectively. All distributions are strongly left-skewed and most of the density falls between 0.8 and 1.0, indicating reviewers consistently favored PK-word2vec. Notably, in the responses from MTurk workers, the sample skew was 1.19, 1.65, and 1.92 for the high-, mid-, and low-frequency words, respectively. In the responses from the medical students, the sample skew was 0.88, 1.46, and 1.84 for the high, mid-, and low-frequency words, respectively. In other words, the degree to which the distribution was left-skewed was inversely proportional to the word frequency.

The results of the mixed-effect model showed that PK-word2vec was significantly more favored than SGNS ($\beta = { }2.702$, p-value = 0.01). By contrast, no difference was found between medical word and non-medical word ($\beta = { }0$, p-value = 1).

## Discussion

This study has several findings that are worth highlighting. First, relying on external word pairs to tune hyperparameters, as has been done in many prior studies^13,27,28^, is suboptimal for smaller datasets. This limitation arises from the fact that many word pairs in external datasets may not be included in the target datasets. To address this issue, we selected hyperparameters by minimizing the distance in the similarity distribution between Google-word2vec and PK-word2vec. Our experiments showed that the prior knowledge weight $\alpha$ changed the word similarity distribution from a unimodal to a bimodal form, suggesting that introducing too much prior knowledge might degrade the quality of word embedding models. However, when comparing the similarity distribution between words across different frequency ranges, we observed that when the prior knowledge weight was very small or very large, it caused the similarity distribution to become dissimilar amongst these word frequency ranges. This finding highlights the necessity of carefully balancing the weight of prior knowledge within the model training.

Second, including context vector regularization into the PK-word2vec training helped reduce the difference in the similarity distribution between words with and without prior knowledge. Specifically, in the model with context vector regularization, the distribution of words with prior knowledge exhibited a longer tail as the value of $\alpha$ increased. By contrast, without context vector regularization, the similarity distribution for words without prior knowledge was right shifted without significant changes to its shape. To investigate the root cause, we inspected the most similar words for those without prior knowledge and located in the tail of the similarity distribution around cosine similarity of 0.9. For example, consider the word *miscarriage*. In the model with context vector regularization, its most similar words were *trimester*, *gestational*, *gestation*, *fetal*, and *fetus*, all of which are directly related to pregnancy. Conversely, without context vector regularization, the most similar words were *tube*, *defects*, *defect*, *homocysteine*, and *suction*. While these terms are associated with miscarriage, they revealed a different pattern of similarity compared to the model with context vector regularization. This effect suggests the importance of context vector regularization in enhancing the model's compatibility with a small sample size of data.

Third, despite using a relatively small dataset, our human reviewers preferred the word groups produced by PK-word2vec over those produced by SGNS, and had a higher support rate for PK-word2vec for non-medical words than medical words. There are several possible explanations for this result. First, the medical words are inherently complex and more difficult for the reviewers to evaluate. Second, the number of medical words was relatively small, such that some medical words are associated with fewer than five other words with high similarity scores. In addition, the new PK-word2vec helped improve the vector representation, especially for low-frequency words (see Fig. 6). While medical students likely had more medical training than MTurk workers on average, our analysis suggested that the two types of reviewers were similar regarding their preferences for non-medical and medical words in this specific survey. But this study is too small to draw definitive conclusions about the broader applicability of this finding. Larger study size and varying experience level of medical experience is needed to explore the feasibility of using a large number of participants pool obtain accurate results for simple medical surveys.

In the context of customizing word representation models, our PK-word2vec method offers a distinct approach compared to previous studies. Several earlier studies have adapted SGNS to enhance word representation, primary focusing on aligning the vector space of a general, pre-trained embedding model to a specific problem domain^29,30^. Other refined strategies included improving the embedding quality by retrofitting the pre-trained word vectors using lexical relational resources^31^, and augmenting SGNS with additional knowledge-based graph models and `anchor context models^32^. Different from these approaches, our PK-word2vec method does not require a large number of training samples to fine-tune the word embedding model. This significant advantage could substantially facilitate research within the field of biomedical informatics, mainly when working with small study cohorts.

Despite the encouraging findings, this study has several limitations that can serve as a basis for future research. First, PK-word2vec was examined solely on patients with breast cancer. It has yet to be determined whether the method generalizes to clinical communications or notes for other types of patients. Second, we generated prior knowledge from two pre-trained models only. It is unknown if incorporating additional pre-trained models can enhance the generalizability of PK-word2vec. Third, complex medical concepts are often represented by long phrases rather than words, which begs the question of how to include more complex concepts in human evaluations. Finally, the model should be tested in downstream NLP tasks by jointly tuning the prior knowledge weight and other hyperparameters.

## Conclusions

This paper introduces PK-word2vec, an adapted word embedding model trained on a relatively small number of patient portal messages. This was achieved by incorporating prior knowledge from two pre-trained embedding models into the model training. Our evaluation with MTurk workers and medical students demonstrated that PK-word2vec outperformed the standard word2vec model in generating more similar words from small-size patient portal messages. While this study focused on extracting prior knowledge from Google-word2ec and Snomed2vec for patient portal messages, the proposed PK-word2vec can leverage prior knowledge from any pre-trained models, irrespective of the vector size or representation space, to build vector representations for other clinical text data.

## Data Availability

The data that support the findings of this study are available on the Vanderbilt University Medical Center Research Derivative, but restrictions apply to the availability of these data due to privacy issue, which were used under license for the current study, and so are not publicly available. Data are however available from the authors upon reasonable request and with permission of Vanderbilt University Office of Research Informatics.

## Code availability

For reproducibility, we open-sourced the Python code for PK-word2vec model at https://github.com/sqyXIU/PK-word2vec.

## References

[1] Dendere, R. *et al.* Patient portals facilitating engagement with inpatient electronic medical records: A systematic review. *J. Med. Internet Res.* **21**(4), e12779. 10.2196/12779 (2019). 30973347 10.2196/12779 PMC6482406

[2] Goel, M. S. *et al.* Patient reported barriers to enrolling in a patient portal. *J. Am. Med. Inf. Assoc.* **18**(1), i8–i12 (2011). 10.1136/amiajnl-2011-000473 PMC3241181 22071530

[3] Kruse, C. S., Bolton, K. & Freriks, G. The effect of patient portals on quality outcomes and its implications to meaningful use: A systematic review. *J. Med. Internet Res.* **17**(2), e44 (2015). 25669240 10.2196/jmir.3171 PMC4342639

[4] Ralston, J. D. *et al.* Patient web services integrated with a shared medical record: Patient use and satisfaction. *J. Am. Med. Inf. Assoc.* **14**(6), 798–806 (2007). 10.1197/jamia.M2302 PMC2213480 17712090

[5] Osborn, C. Y. *et al.* MyHealthAtVanderbilt: Policies and procedures governing patient portal functionality. *J. Am. Med. Inf. Assoc.* **18**(1), i18–i23 (2011). 10.1136/amiajnl-2011-000184 PMC3241162 21807648

[6] Griffin, A., Skinner, A., Thornhill, J. & Weinberger, M. Patient portals. *Appl. Clin. Inform.* **7**(02), 489–501 (2016). 27437056 10.4338/ACI-2016-01-RA-0003 PMC4941855

[7] Yin, Z., Warner, J. L., Chen, Q. & Malin, B. A. Patient messaging content associated with initiating hormonal therapy after a breast cancer diagnosis. In *AMIA Annual Symposium Proceedings, vol. 2019* 962 (2019). PMC7153093 32308893

[8] Yin, Z. *et al.* The therapy is making me sick: How online portal communications between breast cancer patients and physicians indicate medication discontinuation. *J. Am. Med. Inf. Assoc.* **25**(11), 1444–1451. 10.1093/jamia/ocy118 (2018). 10.1093/jamia/ocy118 PMC7646923 30380083

[9] Sulieman, L., Yin, Z. & Malin, B. A. Why patient portal messages indicate risk of readmission for patients with ischemic heart disease. In *AMIA Annual Symposium Proceedings, vol. 2019 *828 (2019). PMC7153079 32308879

[10] Sulieman, L. *et al.* Classifying patient portal messages using Convolutional Neural Networks. *J. Biomed. Inform.* **74**, 59–70. 10.1016/j.jbi.2017.08.014 (2017). 28864104 10.1016/j.jbi.2017.08.014

[11] Glöggler, M. & Ammenwerth, E. Development and validation of a useful taxonomy of patient portals based on characteristics of patient engagement. *Methods Inf. Med.* **60**(1), e44–e55 (2021). 34243191 10.1055/s-0041-1730284 PMC8294937

[12] Wang, Z., Zhang, W., Liu, N. & Wang, J. Scalable rule-based representation learning for interpretable classification. *Adv. Neural Inf. Process Syst.* **34**, 30479–30491 (2021).

[13] Mikolov, T., Sutskever, I., Chen, K., Corrado, G. S. & Dean, J. Distributed representations of words and phrases and their compositionality. In (eds. Burges, C. J. C. *et al*.) *Advances in Neural Information Processing Systems, vol. 26* (Curran Associates, Inc., 2013). https://proceedings.neurips.cc/paper/2013/file/9aa42b31882ec039965f3c4923ce901b-Paper.pdf.

[14] Steitz, B. D. & Levy, M. A. A social network analysis of cancer provider collaboration. *AMIA Annu. Symp. Proc.* **2016**, 1987–1996 (2016). 28269958 PMC5333246

[15] Lochter, J. V., Silva, R. M. & Almeida, T. A. Deep learning models for representing out-of-vocabulary words. In *Lecture Notes in Computer Science (including subseries Lecture Notes in Artificial Intelligence and Lecture Notes in Bioinformatics), vol. 12319* LNAI 418–434 (2020). 10.1007/978-3-030-61377-8_29.

[16] Galea, D., Laponogov, I. & Veselkov, K. Sub-word information in pre-trained biomedical word representations: Evaluation and hyper-parameter optimization. In *Proceedings of the BioNLP 2018 Workshop* 56–66 (2018).

[17] Martinez Soriano, I. *et al*. Snomed2Vec: Representation of SNOMED CT terms with Word2Vec. In *2019 IEEE 32nd International Symposium on Computer-Based Medical Systems (CBMS)* 678–683 (2019). 10.1109/CBMS.2019.00138

[18] Baziotis, C., Pelekis, N. & Doulkeridis, C. DataStories at SemEval-2017 Task 4: Deep LSTM with attention for message-level and topic-based sentiment analysis. In *Proceedings of the 11th International Workshop on Semantic Evaluation (SemEval-2017)* 747–754 (Association for Computational Linguistics, 2017). 10.18653/v1/S17-2126.

[19] Soysal, E. *et al.* CLAMP—a toolkit for efficiently building customized clinical natural language processing pipelines. *J. Am. Med. Inf. Assoc.* **25**(3), 331–336. 10.1093/jamia/ocx132 (2017). 10.1093/jamia/ocx132 PMC7378877 29186491

[20] Mikolov, T., Sutskever, I., Chen, K., Corrado, G. S. & Dean, J. Distributed representations of words and phrases and their compositionality. *Adv. Neural Inf. Process. Syst.* **2013**, 3111–3119 (2013).

[21] Mitra, B., Nalisnick, E., Craswell, N. & Caruana, R. A dual embedding space model for document ranking. arXiv:160201137 (2016).

[22] Grover, A. & Leskovec, J. node2vec: Scalable feature learning for networks. In *Proceedings of the 22nd ACM SIGKDD International Conference on Knowledge Discovery and Data Mining * 855–864 (2016). 10.1145/2939672.2939754 PMC5108654 27853626

[23] Bodenreider, O. The unified medical language system (UMLS): Integrating biomedical terminology. *Nucleic Acids Res.* **32**(1), D267–D270 (2004). 14681409 10.1093/nar/gkh061 PMC308795

[24] Villani, C. *et al.**Optimal Transport: Old and New* (Springer, 2009).

[25] Buhrmester, M., Kwang, T. & Gosling, S. D. *Amazon’s Mechanical Turk: A New Source of Inexpensive, Yet High-Quality Data?* (Springer, 2016). 10.1177/1745691610393980 26162106

[26] Dean, S. & Illowsky, B. *Descriptive Statistics: Skewness and the Mean, Median, and Mode* (Springer, 2018).

[27] Jansen, S. Word and phrase translation with word2vec. arXiv:170503127 (2017).

[28] Chiu, B., Crichton, G., Korhonen, A. & Pyysalo, S. How to train good word embeddings for biomedical NLP. In *Proceedings of the 15th Workshop on Biomedical Natural Language Processing* 166–174 (2016).

[29] Sarma, P. K., Liang, Y. & Sethares, W. A. Domain adapted word embeddings for improved sentiment classification. arXiv:180504576 (2018).

[30] Poerner, N., Waltinger, U. & Schütze, H. Inexpensive domain adaptation of pretrained language models: Case studies on biomedical NER and Covid-19 QA. In *Findings of the Association for Computational Linguistics: EMNLP 2020* 1482–1490 (Association for Computational Linguistics, 2020). 10.18653/v1/2020.findings-emnlp.134.

[31] Faruqui, M. *et al*. Retrofitting word vectors to semantic lexicons. arXiv:14114166 (2014).

[32] Yamada, I., Shindo, H., Takeda, H. & Takefuji, Y. Joint learning of the embedding of words and entities for named entity disambiguation. arXiv:160101343 (2016).

